# Zika and Flavivirus Shell Disorder: Virulence and Fetal Morbidity

**DOI:** 10.3390/biom9110710

**Published:** 2019-11-06

**Authors:** Gerard Kian-Meng Goh, A. Keith Dunker, James A. Foster, Vladimir N. Uversky

**Affiliations:** 1 Goh’s BioComputing, Singapore 548957, Singapore; 2 Center for Computational Biology, Indiana and Bioinformatics, Indiana University School of Medicine, Indianapolis, IN 46202, USA; kedunker@iupui.edu; 3 Department of Biological Sciences, University of Idaho, Moscow, ID 83844, USA; foster@uidaho.edu; 4 Institute for Bioinformatics and Evolutionary Studies, University of Idaho, Moscow, ID 83844, USA; 5 Department of Molecular Medicine, Morsani College of Medicine, University of South Florida, Tampa, FL 33612, USA; vuversky@health.usf.edu; 6 Institute for Biological Instrumentation, Russian Academy of Sciences, Pushchino, Moscow Region 142290, Russia

**Keywords:** Zika, protein intrinsic disorder, shell disorder, dengue, virulence, microcephaly, vaccine, yellow fever, morbidity, fetal

## Abstract

*Zika virus* (ZIKV) was first discovered in 1947 in Africa. Since then, sporadic ZIKV infections of humans have been reported in Africa and Asia. For a long time, this virus was mostly unnoticed due to its mild symptoms and low fatality rates. However, during the 2015–2016 epidemic in Central and South America, when millions of people were infected, it was discovered that ZIKV causes microcephaly in the babies of mothers infected during pregnancy. An examination of the M and C proteins of the ZIKV shell using the disorder predictor PONDR VLXT revealed that the M protein contains relatively high disorder levels comparable only to those of the yellow fever virus (YFV). On the other hand, the disorder levels in the C protein are relatively low, which can account for the low case fatality rate (CFR) of this virus in contrast to the more virulent YFV, which is characterized by high disorder in its C protein. A larger variation was found in the percentage of intrinsic disorder (PID) in the C protein of various ZIKV strains. Strains of African lineage are characterized by higher PIDs. Using both in vivo and in vitro experiments, laboratories have also previously shown that strains of African origin have a greater potential to inflict higher fetal morbidity than do strains of Asian lineage, with dengue-2 virus (DENV-2) having the least potential. Strong correlations were found between the potential to inflict fetal morbidity and shell disorder in ZIKV (*r*^2^ = 0.9) and DENV-2 (DENV-2 + ZIKV, *r*^2^ = 0.8). A strong correlation between CFR and PID was also observed when ZIKV was included in an analysis of sets of shell proteins from a variety of flaviviruses (*r*^2^ = 0.8). These observations have potential implications for antiviral vaccine development and for the design of cancer therapeutics in terms of developing therapeutic viruses that penetrate hard-to-reach organs.

## 1. Introduction

The *Zika virus* (ZIKV) was first isolated in 1947 when three owl monkeys from the Zika Forest, Uganda, were found to have a fever [1,2]. Even though the first human case was reported in 1952 and although this virus has been circulating in Asia and Africa, very few ZIKV cases were noticed and recorded before the 2015/2016 epidemic, since the symptoms were generally mild [1,3] and fatality was rare. In 2014, the first sign of an oncoming ZIKV epidemic appeared in French Polynesia with around 35,000 known cases of infection. This was followed by a full-blown epidemic in Latin and Central America in 2015/2016, with approximately 1.5 million estimated cases in Brazil alone [3,4]. With such a large number of infections, physicians were able to notice exceptionally large numbers of microcephaly cases among the babies of women who were infected by ZIKV during their pregnancies [1,3,5,6]. Microcephaly refers to a condition in which a baby is born with a smaller than usual brain and head. Further studies of ZIKV in laboratories using animal models confirmed the ability of ZIKV to penetrate both the placenta and the brain [7,8,9,10,11,12,13].

ZIKV is a member of the genus *Flavivirus*, which encompasses a large variety of arthropod-borne enveloped RNA viruses, including dengue virus (DENV), West Nile virus (WNV), and the notoriously pathogenic yellow fever virus (YFV). Similarly to YFV and DENV, ZIKV is also spread by the mosquito, *Aedes aegypti* [2,6,14]. A flavivirus genome encodes a polyprotein that includes 10 proteins, three structural proteins (capsid (C), precursor membrane (PrM), and envelope (E) proteins), and seven nonstructural (NS) proteins (NS1, NS2a, NS2b, NS3, NS4a, NS4b, and NS5) [14,15]. The immature virion contains an abundance of PrM protein, which is cleaved during maturation, and only M remains in a mature virion [16,17]. In a mature virion, the outermost shell contains E protein, which is followed by a shell containing M and C proteins. In an immature virion, however, both E and PrM are contained in the outer shell [16,17].

The results presented in this study pertain to the concept of protein intrinsic disorder, which is related to proteins or protein segments that have no ordered structure, but this lack of structure can be related to the function of these proteins [18,19,20,21]. Such proteins are often known by other names, such as “natively unfolded” and “intrinsically unstructured” proteins [20,22]. The penetrance of disorder in the proteins from flaviviruses and ZIKV has already been analyzed [23,24,25]. The abundance and specific functionality of intrinsically disordered proteins in viral proteomes in general have been considered in several large-scale computational studies [26,27] and have been specifically analyzed for human papillomaviruses [28,29], different strains of the influenza virus [30], human immunodeficiency virus type -1 (HIV-1) [31], human hepatitis C virus [32,33], *Dengue virus* [34], respiratory syncytial virus [35], *Chikungunya virus* [36], and *Alkhurma virus* [37]. Furthermore, previous studies have found correlations between virulence and the shell disorder of DENV and flaviviruses, although the sets used in those studies did not include ZIKV proteins [2,14]. The shell proteins examined were M and C proteins, and the highly pathogenic YFV was found to contain the highest levels of shell disorder among the fairly large variety of flaviviruses looked at. Although ZIKV shell disorder has also been inspected in other studies, a comparative analysis between flaviviruses and ZIKV strains with respect to shell disorder and virulence has not been conducted as of yet. Intriguingly, while ZIKV is not known for its virulence [5], there are distinct disparities in the levels of potential to cause fetal morbidity between various ZIKV strains of different lineages [38,39]. However, no attempt has been made to correlate fetal morbidity and shell disorder until now. The research conducted in this paper demonstrates not only that ZIKV fits well within the previously observed correlations between flavivirus virulence and shell disorder, but that the uniqueness of the ZIKV shell disorder pattern is consistent with the varying levels of fetal morbidity caused by strains of different lineages.

## 2. Materials and Methods

The appropriate proteins and their sequences were carefully searched and checked upon retrieval from UniProt (http://www.uniprot.org) or NCBI (http://www.ncbi.nlm.nih.gov/structure/). After downloading the sequences and respective protein data bank (PDB) files, the necessary information was imported into the database using tools implemented in JAVA [40]. The corresponding protein sequences in FASTA format were fed into the PONDR VLXT neural network (http://www.pondr.com). The results were given as scores from 0 to 1. Residues with scores of 0.5 and over represent disordered residues [41,42]. An important ratio used in this and previous studies is the percentage of intrinsic disorder (PID), which is defined as the number of residues predicted to be disordered in a protein chain divided by the total number of residues in the chain. The PID can range from 0% (perfectly ordered) to 100% (totally disordered).

The predicted intrinsic disorder-related information was retrieved and fed into a MYSQL database that has been mentioned in previous papers [40]. Another JAVA-DBI program then automatically created codes that were readable in Jmol (www.jmol.com), which generated a 3D representation of protein crystal structures with disorder annotations that could be visualized from the colors provided by the codes. The Jmol software also required the respective PDB (protein data bank) files, which could be obtained from NCBI, as mentioned above.

A PONDR VLXT predictor was used in this study, since it is one of the most informative tools for structural proteins, namely for viral shells, where protein–protein interactions play vital roles in order–disorder transitions [41,42,43,44]. It has successfully been utilized to study the shell disorder of a large variety of viruses, including HIV, smallpox, and polio viruses [2,14,25,30,45,46,47,48,49,50,51,52]. PONDR represents a suite of computational tools for sequence-only predictions of the intrinsic disorder predisposition of query in proteins. PONDR VLXT was the earliest predictor created at the end of the last century [41,42]. It involves the use of neural networks that are trained to recognize protein sequences that are ordered and those that are not [53]. Statistical methods, including regression analysis and analysis of variance (ANOVA), were used to analyze the PONDR VLXT results along with data on virulence and morbidity. The statistical package R version 3.6.0. [54] was used to obtain the results. Before obtaining the necessary results, the respective codes had to be written in R.

## 3. Results

### 3.1. The Correlation between Shell Disorder in Flaviviruses and Zika and Virulence and Fetal Morbidity

**Figure 1:**
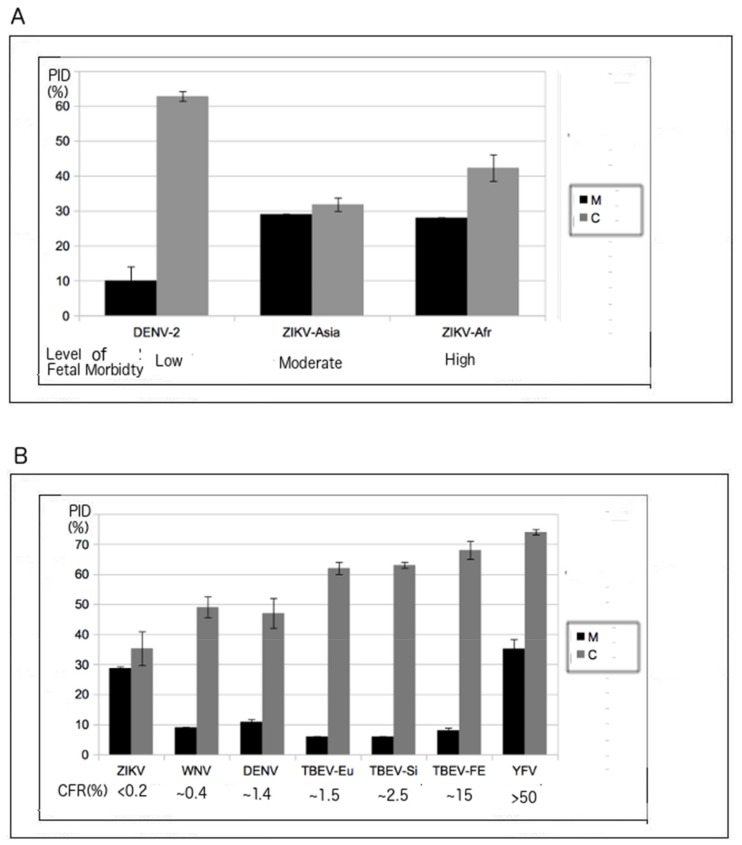
Correlations between fetal morbidity/mortality and shell disorder (M and C Protein PIDs). (**A**) Correlation between fetal morbidity and ZIKV–*Dengue virus* 2 (DENV-2) shell disorder (regression: *r*^2^ = 0.8, *F* = 16, *p* < 0.01; independent variables: C and M PIDs). See Figure S1 for more details on the data points; (**B**) correlation between flavivirus case fatality ratio (CFR) and shell disorder (regression: *r*^2^ = 0.8, *F* = 29, *p* < 0.01; independent variables: C and M PIDs). With the exception of the ZIKV data, much of the data can be found in a previous publication [25].

While ZIKV is not known for its virulence, fetal morbidity, as already mentioned, is a threat from ZIKV infection [1,2,3,6]. ZIKV strains are generally categorized as of African or Asian lineage, and the epidemic that occurred in 2015–2016 involved Asian strains that had crossed the Pacific Ocean to reach the Americas. At least two laboratories have found that ZIKV strains of African lineage are more dangerous to fetuses than those of Asian lineage [38,55]. The classical isolate for the strains of African lineage is MR-766, which was discovered in Uganda in 1947 [1,56]. A PID-based wide selection of ZIKV strains and isolates allowed us to see that the strains can be divided into four groups that are consistent with their lineages but that are independent of the dates of the isolates (Figure 1A and Table 1). Interestingly, our results show that there are at least two MR-766 isolates from Uganda-1947 with distinct shell disorder features (Table 1). Furthermore, DENV-2 has also been shown to cause microcephaly in fetuses and newborns, but to a lesser extent when compared to ZIKV [38,39,55]. This has been observed in several laboratories using in vivo and in vitro methods. Chicken liver cells, human nerve cells, and mouse models were used, and the levels of destruction by ZIKV and DENV-2 were observed according to the amount of cells destroyed [38,39,55]. The PIDs of DENV-2 and ZIKV are compared in Figure 1 and Table 1.

Various statistical methods were used to reaffirm not just the statistical significance of the grouping by shell PID (especially the C protein), but also the statistical significance of a strong correlation between the PID of the C protein and fetal morbidity, given the evidence of greater fetal morbidity in infections by MR-766. Regression analyses and an ANOVA conducted on the data shown in Table 1 provided evidence that the shell PID differences between strains of the African and Asian lineages are statistically significant (Table 1; regression: *r*^2^ = 0.9, *F* = 15, *p* < 0.01; one-way ANOVA with C PID as the independent variable: *F* = 53, *p* < 0.01). When data related to DENV-2 were added to the analysis, a strong correlation that was statistically significant was found (*r*^2^ = 0.8, *F* = 16, *p* < 0.01).

While Figure 1A and Table 1 involve the determination of the correlation between the fetal morbidity associated with ZIKV infection and ZIKV shell disorder (PID), Figure 1B and Table 2 represent the results of the analysis of the correlation between the case fatality ratio ((CFR), i.e., virulence) and the shell disorder of various flaviviruses, including ZIKV. Information on the CFRs of various flaviviruses is publicly available [14,57,58,59,60]. Because the symptoms of ZIKV are usually very mild and fatalities are extremely rare, for a long time, the virus was shrouded in mystery [2,3].

It is therefore likely that the CFR is very close to zero, even if most of the currently available data are those from infections arising in the Americas, which involved strains of Asian lineage, not African lineage [1,4,5,6]. A strong correlation was found between flavivirus CFR and shell disorder, as seen in Figure 1B and Table 2. While a correlation between flavivirus CFR and shell disorder has already been reported [2,14], the data presented here include results for ZIKV, which was not previously considered.

### 3.2. C and M Proteins Play Roles in Virulence and Morbidity

In a previous study, we established that both C and M proteins play roles in flavivirus virulence, as represented by CFR [25]. According to our research on other viruses, the capsid association with virulence involves a mechanism of protein promiscuity, whereby intrinsic disorder allows the capsid protein (the C protein, in the case of flaviviruses) to bind to a greater number of proteins, thereby allowing the virus to multiply more rapidly before the host immune system can take action [2,14,51]. The matrix protein (the M protein in flaviviruses), on the other hand, is likely to play a somewhat different role. A more disordered matrix allows the virus to penetrate organs, namely vital organs, as in the case of HIV-1, but still be able to evade the host immune systems via matrix and surface glycoprotein motions [14,46,52].

A closer look at Table 1 and Table 2 and Figure 1 reveals other specific trends. As in previous studies, the PID of the C protein, as the sole independent variable, provided a weaker correlation with flavivirus CFR that was of statistical significance (*r*^2^ = 0.3, *F* = 6, *p* < 0.05). Similarly, the PID of the M protein, as the sole independent variable, also did not provide a very strong correlation with flavivirus CFR, even if this correlation was statistically significant (*r*^2^ = 0.4, *F* = 9, *p* < 0.05). The use of PIDs of both M and C proteins as independent variables, on the other hand, gave a strong correlation with flavivirus CFR that was definitely stronger than the PIDs of C or M proteins alone (*r*^2^ = 0.38, *F* = 14, *p* < 0.01, Figure 1B). Obviously, both M and C proteins complement each other in their combined contributions to virulence, which can be seen in both Figure 1B and Table 2. There was also a statistically significant interaction between the two factors (*p* < 0.01).

### 3.3. Regions of Disorder in the M Protein: ZIKV versus YFV and DENV-2

**Figure 2:**
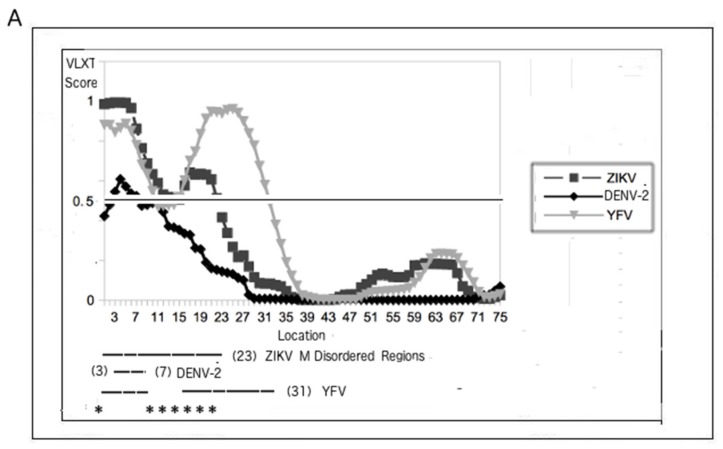

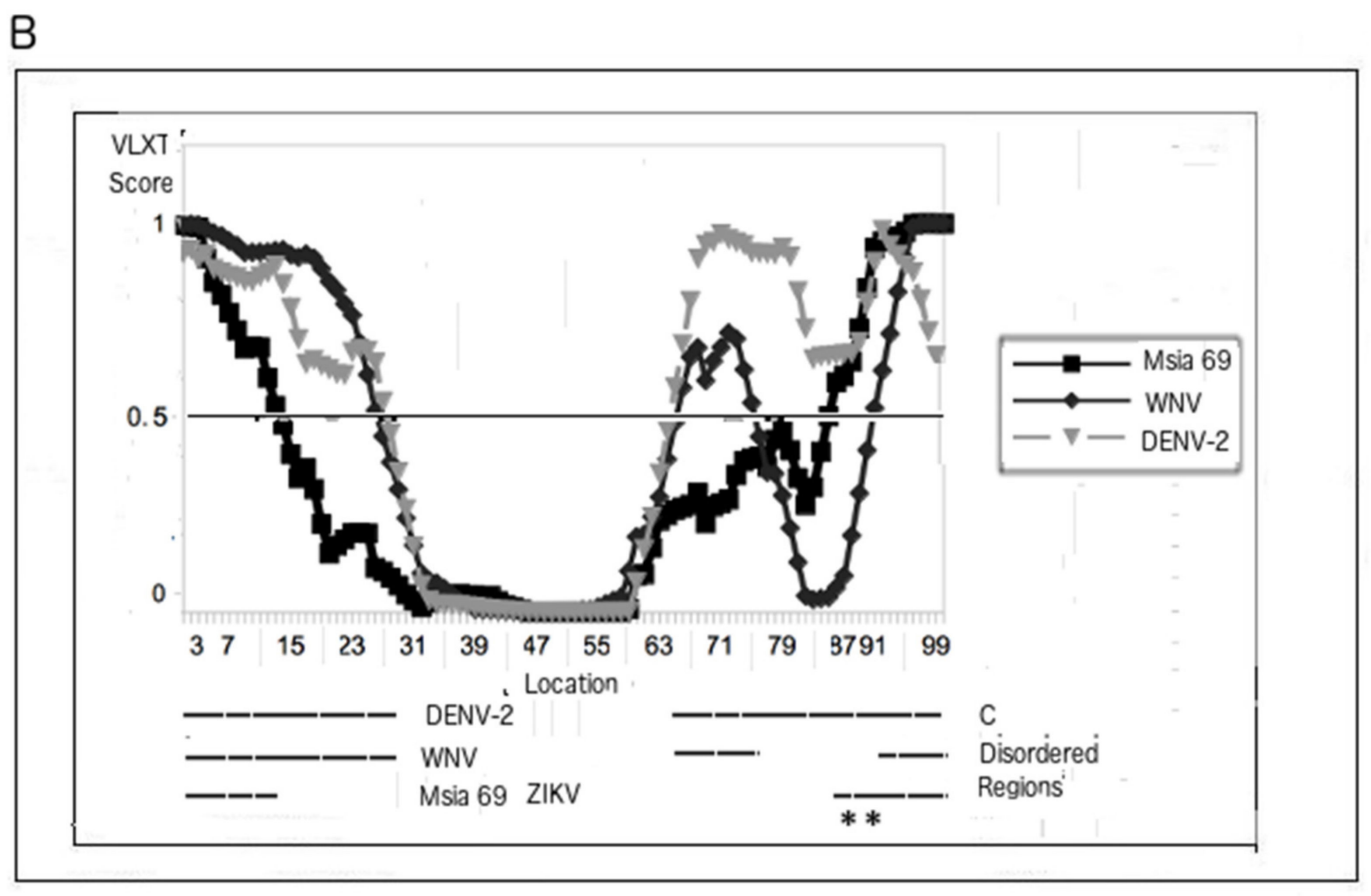
Comparative PONDR VLXT plots of the M and C proteins from various flaviviruses, including ZIKV. (**A**) M proteins of ZIKV (UniProt: A0A127AM58), DENV-2 (UniProt: P29990), and YFV (UniProt: Q1X881). (**B**) C proteins of ZIKV (UniProt: H8XX11, Msia69, see Table 1), WNV (UniProt: Q9Q694), and DENV-2 (UniProt: P29990). Regions with scores of 0.5 or above represent disorder. Disorder differences between C proteins can be traced mainly to mutations and disorder near the N- and C-termini. The regions with an asterisk (*) denote potential targets for the development of a ZIKV vaccine. With the exception of Msia69 (Malaysia 1969) ZIKV, the strains were randomly chosen. Msia69 was deliberately chosen as a representative Asian strain that has a low C PID. The YFV M protein was chosen for (**A**), since it has one of the highest PIDs among flaviviruses. WNV was not necessary for (**A**), as specific strains of DENV-2 have an even lower M PID (PID: 6%) than does the ZIKV (PID: 9%). On the other hand, WNV was chosen for (**B**), as the low WNV C PID (among flaviviruses) allows us to suggest strategies for ZIKV vaccine development.

**Figure 3:**
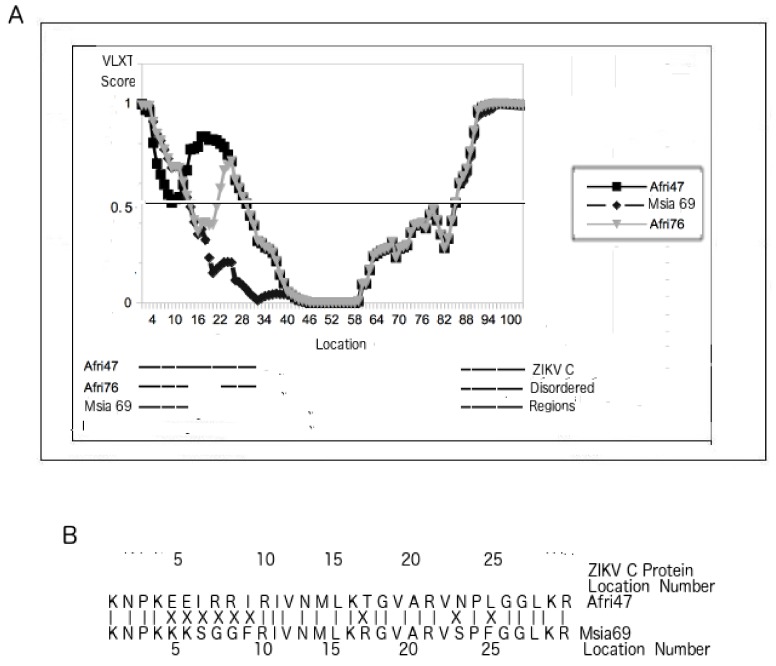
(**A**) Comparison of intrinsic disorder predisposition of the C protein in ZIKV strains. PONDR VLXT plot of C proteins from different ZIKV strains. (**B**) Mutations and disorder of the N-terminus of the C proteins of the Afri47, Afri76, and Msia69 (the Africa 1947, Africa 1976, and Malaysia 1969 strains, respectively; Afri47 is the ZIKV/*Macaca mulatta*/UGA/MR-766/1947-East_African isolate; see Table 1 for further details) strains. Disorder differences between C proteins from the Asian and African lineages can be traced mainly to mutations and disorder near the N-termini.

Figure 1 and Table 1 and Table 2 demonstrate that ZIKV is a peculiar virus, since it has a relatively disordered M protein but also a relatively ordered C protein. This accounts for the ZIKV characteristics of low mortality rates and yet high fetal morbidity rates. However, the use of PID values is inadequate in providing a closer look at the differences in the disordered regions among the various viruses. Figure 2 and Figure 3 provide us with the opportunity for a detailed look at the disorder differences by region of the proteins among several flaviviruses.

Figure 2A shows that disordered regions of the M proteins of ZIKV and YFV are relatively large and overlap. With the disordered regions being found mostly around the N-termini of the M proteins of the three flaviviruses shown, a much shorter disordered region is found in the DENV-2 M protein. These observations could be used as a template for designing a ZIKV vaccine, especially when looking at the PONDR VLXT plot of the ZIKV M protein. The asterisk (*) in Figure 2 shows areas that could be potential targets in the development of a ZIKV vaccine.

### 3.4. PONDR VLXT Plot for C Proteins from ZIKV and Other Flaviviruses

The PONDR VLXT plot for the C proteins of three flaviviruses, ZIKV, DENV-2, and WNV, can be found in Figure 2B. As mentioned, studies have shown that disorder in the inner shells of viruses in general tend to be associated with greater virulence because of the ability of proteins to bind more promiscuously, thus providing the viruses with greater ability to replicate more rapidly. Similarly, Figure 1B and Table 2 suggest that the reason that the ZIKV’s CFR is extremely low has to do with the relatively low mean PID of its C protein (35 ± 6%, CFR ~0). While these results tell us about the differences with regard to the mean C PIDs among the various flaviviruses, they do pinpoint the disorder differences among the various flaviviruses along the entire stretch of the various flavivirus C proteins, especially with respect to ZIKV. Figure 2B attempts to address this problem by showing that the disorder differences between C proteins from ZIKV and other flaviviruses lie in two areas near the N- and C-termini, respectively.

### 3.5. PONDR VLXT Plot for C Proteins from Different ZIKV Strains

While Figure 2B, Figure 1B, and Table 2 provide evidence that disorder in the C protein contributes to the virulence of flaviviruses, Figure 1A, Figure 2A, Figure 3, and Table 1 point to the contribution of ZIKV C protein disorder to fetal morbidity. More specifically, a rather large disordered region is missing near the N-terminus of the strains of Asian lineage, which, as already mentioned, inflict morbidity to fetuses at a lower rate than do their African counterparts. There are also two distinct patterns of disorder for the two separate groups of MR-766-like strains (ZIKV/*Macaca mulatta*/UGA/MR-766-VEROE6-AC4-P12_07/1947 vs ZIKV/*Macaca mulatta*/UGA/MR-766/1947/East_Africa) shown in Table 1.

While Figure 3A reports that the residues that are disordered only in the MR-766-related strains can be found near the N-terminus, Figure 3B identifies the residues responsible for the disorder differences. The residues marked with “X” represent mutations. In general, the more polar and charged residues tend to induce more disorder, and such can be observed in this case. Disorder differences between C proteins from the Asian and African lineages can be traced mainly to mutations and disorder near the N-termini.

### 3.6. The Roles of M Versus PrM and Pr in Protecting the Virion at Various Stages

The structure of immature flaviviruses is somewhat different from mature ones. An immature flavivirus virion includes a precursor membrane protein, PrM. During maturity, the PrM is cleaved such that the M remains as the membrane protein. This characteristic raises important questions about the roles of PrM and M proteins, especially with regard to protecting the virion.

**Figure 4:**
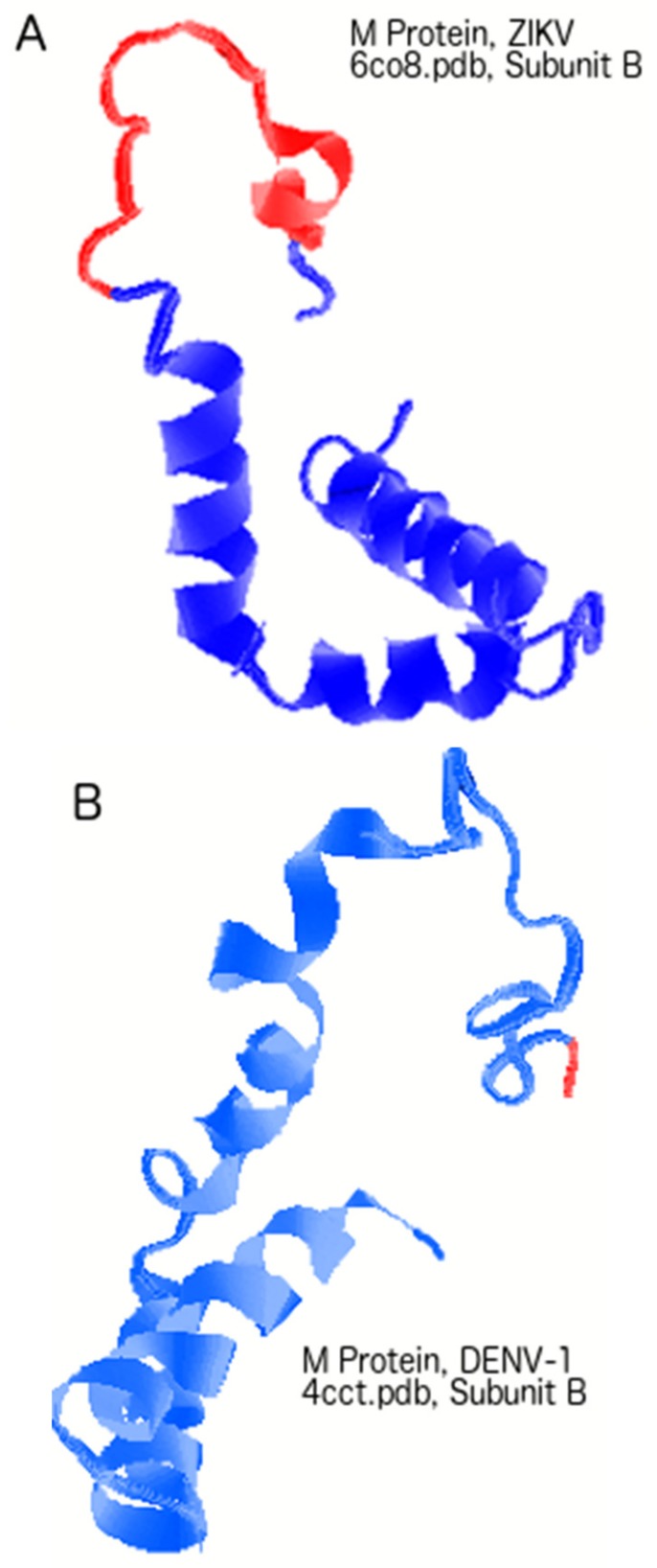

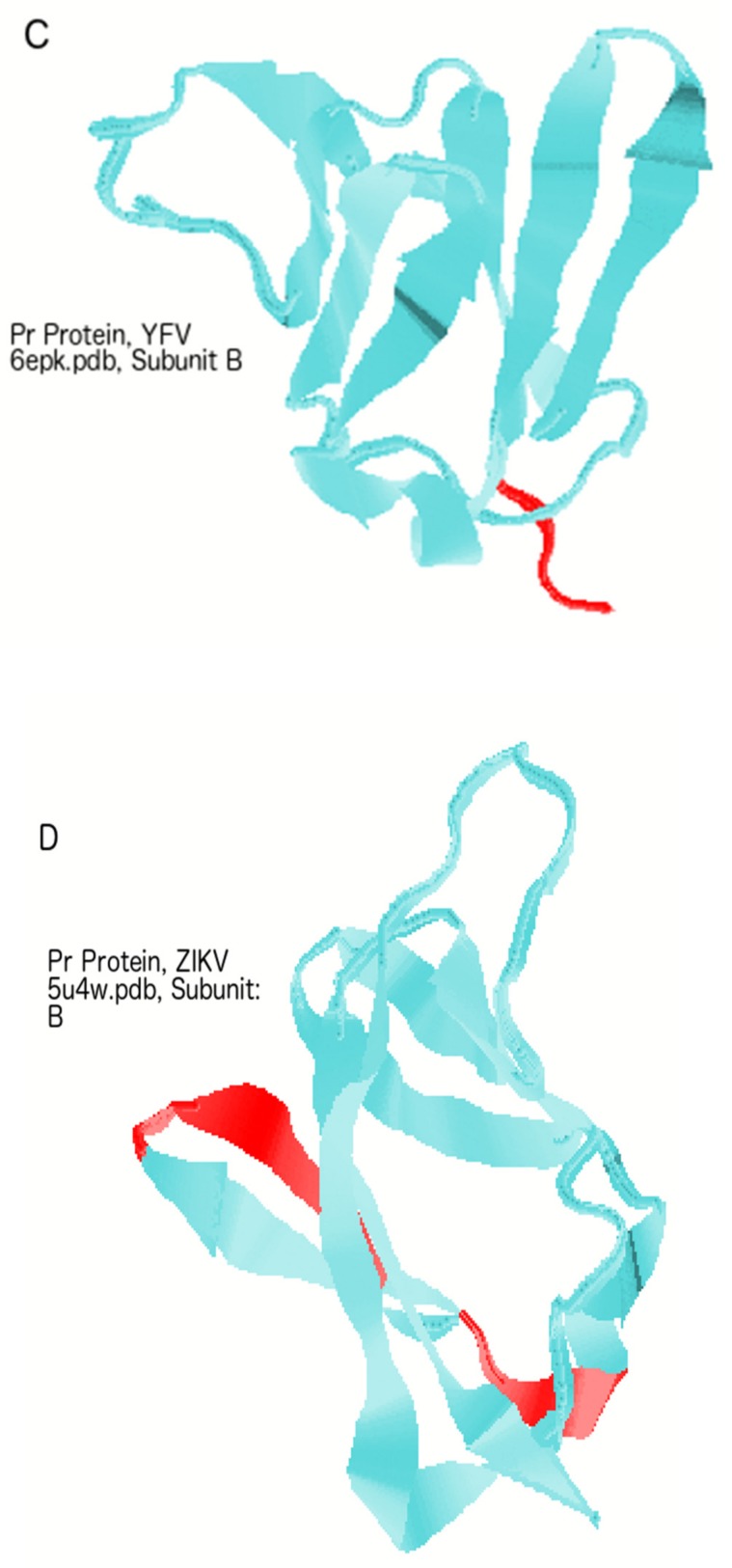

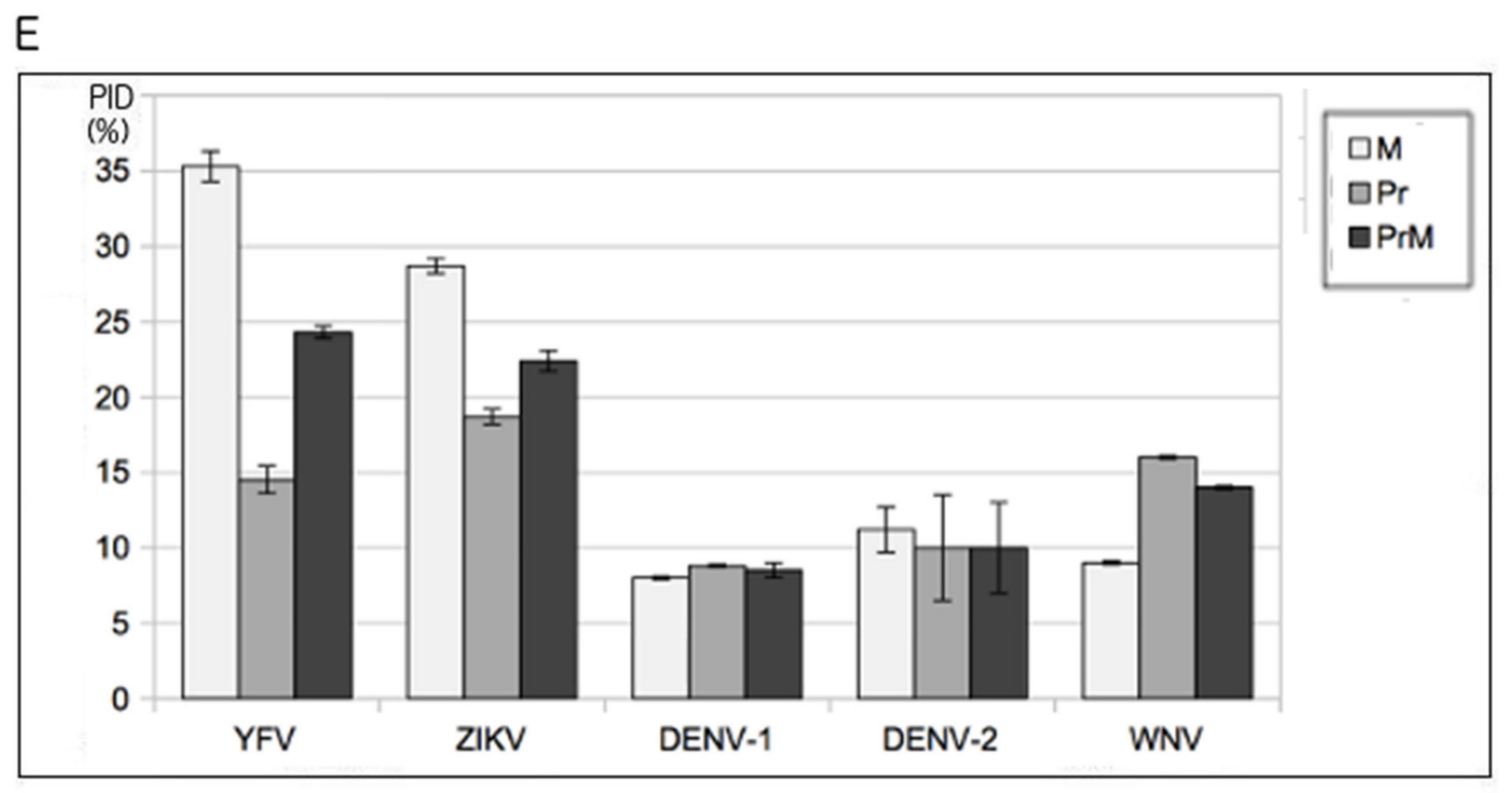
A comparison of M and PrM proteins (membrane and precursor membrane proteins) among the various flaviviruses. (**A**) Three-dimensional structure of the ZIKV M protein, with disordered regions represented in red. (**B**) Cryoelectron microscopy structure of the DENV-2 M protein (with disorder annotation). (**C**) Crystal structure of YFV Pr, with disorder annotated in red. (**D**) Cryo-EM (cryogenic electronic microscopy) structure of ZIKV Pr, with disorder annotated in red. (**E**) A comparison of Pr, M, and PrM among the various flaviviruses. PrM and M proteins protect the immature and mature virions, respectively.

While such roles have seldom been explored in the past, hints of the potential roles of PrM and M proteins can be found in the PID values obtained in this study. We have seen that with the exception of YFV and ZIKV, most other flaviviruses have highly ordered M proteins to protect their respective mature virions (Figure 1B and Table 2). Figure 4A shows that the M protein is relatively more disordered (in red) than in DENV-1 (Figure 4B). As with the PONDR VLXT plots in Figure 2, the disordered regions of both ZIKV and DENV-1 are restricted to segments near the N-termini. While we have seen that the M proteins of ZIKV and YFV are relatively disordered, the same cannot be said about Pr. The Pr proteins for YFV and ZIKV are quite ordered, as seen in Figure 4C–E. The Pr disorder levels are relatively low for most flaviviruses [2,25], as seen in the cases of ZIKV, DENV, and WNV. With Pr being mostly ordered for most flaviviruses and M being the same for most flaviviruses (with the exception of YFV and ZIKV), one can conclude that both Pr and M contribute to the order of the PrM proteins for most flaviviruses, and this is especially important with respect to protecting the immature virion.

The following question then arose: How do ZIKV and YFV protect their immature virion given their abnormally high M disorder? A hint to the answer to this question lies in the PID of their Pr proteins. As we can see in Figure 4C–E, the Pr proteins of both ZIKV and YFV are quite ordered, well within the range of most flaviviruses. As a result, fairly ordered PrM proteins are seen in both ZIKV and YFV. The study therefore shows that the immature virions of ZIKV and YFV are more protected against environmental insults than are their corresponding mature virions, which could suggest a slightly different life cycle for YFV and ZIKV.

While mature viruses are of course infectious, experimental evidence has suggested that fully immature flaviviruses are generally not infectious, even if they may become infectious upon interactions with antibodies [61]. Partially mature viruses are likely more infectious than fully immature ones [62]. ZIKV and YFV could therefore rely more on lesser mature viruses for their spread, given our evidence showing that their virions become more delicate upon maturation.

## 4. Discussion

### 4.1. Virulence and Disorder from Inner and Outer Shells: Clues from Other Viruses

The data in Figure 1, Figure 2, Figure 3 and Figure 4 and Table 1 and Table 2 show correlations between the intrinsic disorder predisposition of proteins from the inner and outer shells and mortality and fetal morbidity rates. This flavivirus-wide statistical study shows that both M and C proteins play roles in pathogenesis. Similarly, the statistical analyses reveal that disorder in both the C and M proteins also contributes to fetal morbidity.

### 4.2. A Lesson from HIV: Disordered Outer Shell Provides Better Viral Penetration of Vital Organs

The regression analysis reported here suggests the presence of interaction effects between C and M PIDs. Given these observations and the fact that similar proteins play similar roles even among nonrelated viruses, what do other viruses have to offer with respect to clues about trends in the correlation between virulence and shell disorder? While very few viruses have shown positive correlations with statistical significance between their virulence and membrane/matrix disorder (as the sole independent variable), HIV offers a unique insight into the role of the matrix (membrane) protein. Of the HIV and simian immunodeficiency virus (SIV) family, the HIV-1 matrix has the greatest known PID [46,52]. HIV-1 and its closest sibling, SIVcpz, are the most pathogenic when compared to their other cousins and siblings, including HIV-2. HIV-1 patients are known to have much greater viral loads in their bloodstream than patients with HIV-2. Without the help of retroviral drugs, over 90% of HIV-1 patients would die within 10 years of the infection, whereas those infected with HIV-2 take a much longer time to succumb to the disease, if at all [46,63]. The roles of the matrix protein in virulence have to do with viral ability to evade the host immune system and also the ability to bind promiscuously to more host proteins [18,19,21,22]. An important characteristic of HIV is its ability to penetrate vital organs, including the brain, with ease [14]. Apparently, the promiscuous binding potential arising from the disordered matrix shell allows HIV to have easier access to specialized cells. We shall use this argument again in the cases of ZIKV and YFV, which have high PIDs for their M proteins (28.6 ± 0.5% and 35 + 1%, respectively).

### 4.3. Virulence and Inner Shell Disorder Involve a Different “Trojan Horse” Strategy in Immune Evasion

While disorder in the matrix shell is associated with virulence and the immune evasion found in HIV, disorder in the inner shell (capsid or nucleocapsid) of other viruses, such as *Ebola virus* and *Nipah virus*), is often also strongly associated with virulence [45,51]. Virulence arising from disorder in the inner shell involves a different strategy of immune evasion, which can be referred to as Trojan horse, since the virus attempts to replicate rapidly before the host immune system is able to recognize its presence [2,14]. It does so by using the disordered inner shell proteins that play important roles in the replication of the virus, where disorder assists the process by allowing for greater promiscuous (and therefore more efficient) binding of host proteins.

### 4.4. Taking Turns to Protect the Virion from Environmental Insults

Curiously, a conflicting trend has also been observed in HIV: when the outer shell is disordered, then the inner shells tend to be more ordered [14,52]. Conversely, if the outer shell is more ordered, the inner shells tend to be more disordered. These conflicting trends can be seen in a variety of viruses, including Nipah, corona, and Ebola viruses, not just HIV [2,45,49,50,51]. The reason for this has to do with the fact that both shell layers play their roles in protecting the virion from environmental insults. If the outer layer is more disordered, the inner layer compensates by being more ordered. If, on the other hand, the outer layer is more ordered, the inner layer, depending on the type of environment the virus is exposed to, has the luxury of being less ordered.

### 4.5. Conflicting Trends Can Be Found among Flaviviruses: Protecting the Virion Versus Immune Evasion

The two aforementioned seemingly conflicting trends that are seen in other viruses can also be observed in flaviviruses and ZIKV. It is for this reason that we observed statistical significance in the interaction effects between the PIDs of C and M proteins as independent variables. Furthermore, strong flavivirus CFR correlations were not attainable until both C and M PIDs were used as independent variables (*r*^2^ = 0.8, *F* = 29, *p* < 0.01). A closer look at the data in Figure 1B allows us to conclude that some flaviviruses do indeed have more ordered M proteins when their C proteins are disordered and vice versa. For example, tick-bone encephalitis virus (TBEV) viruses have higher PIDs in their C proteins than do DENV viruses, but they have lower PIDs for M proteins. Similarly, ZIKV has a low PID value for C but a high PID for M. There is also evidence of this trend within ZIKV strains themselves: we can notice a slightly lower M disorder in MR-766 strains (PID: 28 vs 29%, Table 1) despite their higher C PID values even as M PIDs remain relatively high for all ZIKV strains.

This trend is, however, not the case with every flavivirus. A striking exception is YFV, which provides some evidence for the other trend that involves strategies of immune evasion using disorder in either or both M and C shells. More specifically, disorder in M and C proteins is likely to play different but complimentary roles in maintaining higher viral load, especially in vital organs necessary to evade the immune system, and YFV is somewhat unique in using both C and M disorder to a much greater extent than other flaviviruses do. This will be further described in the next section.

### 4.6. YFV Uses both Inner and Outer Shell Disorder to Evade Immune Systems, Resulting in High Pathogenicity

Relative to other flaviviruses, YFV is characterized by highly disordered C and M proteins. Two modes of action are at work here. A highly disordered YFV has an overwhelmingly high PID in its C protein (~75%). Interestingly, the mean PID of its M protein (~35%) was also far higher than the corresponding values for all other flavivirus species found in our sample. It is likely that the highly disordered C protein allows for more rapid replication via its promiscuity in protein binding. As a result of the rapid replication, greater viral loads are more attainable, leading to greater virulence. A higher disorder in the M protein, however, allows the virus to more easily penetrate different organs, including vital organs such as the liver [2,15]. Apparently, YFV’s extremely high pathogenicity, which reaches above a 50% CFR, arises from its unique ability to utilize disorder from both its M and C proteins to maintain a viral load as a means of evading the immune system.

### 4.7. The Shell Disorder Model Predicts Correctly that ZIKV Is Able to Penetrate Vital Organs with Greater Efficiency

While YFV has greater disorder in both its M and C proteins, ZIKV offers us a unique opportunity to study a flavivirus with a high PID in its M protein (35 ± 6%) but a low PID in its C protein (28.6 ± 1%). If we use the aforementioned model based on our knowledge of shell disorder, the model will predict ZIKV as a virus that is of low pathogenicity but has the ability to penetrate vital organs. This prediction is extremely accurate in the case of ZIKV. We are able to see that ZIKV infection is usually nonfatal, with CFR being close to 0%, but ZIKV also has an uncanny ability to penetrate the brain and placenta, as seen in its ability to cause microcephaly [12].

### 4.8. ZIKV C PID Variation Correlates with Fetal Morbidity: A “Trojan Horse”

We have seen that flaviviruses use disorder in M and C proteins to evade host immune systems. YFV uses both M and C to penetrate vital organs and proliferate rapidly. The two modes become a deadly mix. The ZIKV uses its high M disorder to penetrate the placenta and brain, but we have also noticed that, while ZIKV C PID values are generally low, there is noticeable variation in the C PIDs seen in the different lineages and strains. This PID variation in the C protein correlates with fetal morbidity that is dependent on the lineage of ZIKV. The results fit perfectly with what has been observed in other viruses. The “Trojan horse” theory tells us that the higher C disorder allows for more efficient proliferation of the virus in the larger variety of organs that it is able to penetrate [2,49].

### 4.9. Evolution of ZIKV Variation in Fetal Morbidity Is Dependent on the Optimal Viral Load Necessary for More Efficient Transmission Among Its Primary Primate Hosts

While variation in ZIKV M PID is only 1%, ZIKV C PID can be as low as 31% and as high as 45%. Inspection of the PONDR VLXT plot shows that the disordered regions lie in both the N- and C-termini even if the ZIKV C mutations arise entirely within the N-terminus (Figure 3). This provides clues to the evolution of ZIKV with respect to its ability to cause fetal morbidity in its hosts. It is likely that ZIKV had to evolve with the primate species that it most commonly infects. For certain primate hosts, it finds greater fitness when a greater viral load is necessary for it to infect a larger number of hosts because of the peculiarities found in the hosts’ immune system: having a more disordered C protein does the trick. It is therefore likely that the different PIDs of C proteins from the various ZIKV strains and lineages are the result of the types of primates that the peculiar ZIKV strain primarily infects and its ability to fine-tune its C disorder to meet the optimal viral load necessary for more efficient transmission of the virus in a given host, as seen in the case of other viruses and other flaviviruses [2,14,25,39,49,50].

### 4.10. Experimental Evidence for the Role of C and M Proteins in Replication and Immune Evasion: Roles for Disorder

Experiments do provide evidence pointing to the important roles of C and M proteins in replication and immune evasion. Flavivirus protein C, along with other viral proteins, assists in the assembly and packaging of viral particles during replication, in addition to assisting the protein M to move to the endoplasmic reticulum (ER). Additionally, the C protein is trafficked to the nucleus through binding with importin [64]. Upon doing so, it interacts with histones to force the cell to stay longer in the G1 stage of the cell cycle so as to buy more time for viral replication [65]. In the cytoplasm, protein C binds to the Sec3 protein in order to inhibit Sec3 proteasomal antiviral activity [66].

As for the M protein, there is still much to be uncovered about its functions. It is, however, known that PrM binds to the ER for two reasons. It protects the E proteins from damage by host enzymes via the formation of a beta barrel [67]. The other reason is that PrM prevents E from being so embedded in the host membrane that it cannot be removed in the later stages of the virion replication process. While the Pr segment is ordered to protect E from the proteases, the greater flexibility of M in many flaviviruses is likely needed to ensure greater efficiency in its binding to the ER.

Similarly, greater disorder in the C protein offers plenty of opportunity for greater binding efficiency, given its aforementioned experimentally proven functions. For instance, the greater disorder in the C protein could help its binding to histones with greater efficiency. Protein disorder not only provides greater efficiency in protein–protein binding but also promotes the binding of proteins to nucleic and fatty acids as well.

### 4.11. Clues for Developing a ZIKV Vaccine

Figure 3 reiterates the minimum requirements necessary in the development of a ZIKV vaccine: the PID of the C protein has to be as low as possible. Figure 2B, on the other hand, suggests ways that a ZIKV can be used for a ZIKV vaccine developed by comparing the PONDR VLXT plots of the ZIKV C protein to those of other flaviviruses. More specifically, it shows a region near the C-terminus that is ordered in WNV but disordered in ZIKV. This region could be a good target for the development of an attenuated ZIKV, as it indicates a region that is not necessarily disordered but is still tolerated by a cousin, WNV.

Just as relatively high disorder levels in the M protein (PID: 28%, 29%) are a hallmark of the ZIKV, an effective vaccine needs to have an M protein with a lower PID so that an attenuated virus will not penetrate the placenta of pregnant women. Figure 2A and Figure 4 show that the disordered regions of the M protein are near the N-terminus, and mutations to attenuate the virus must be in that region. Since the M protein of DENV has a much shorter disordered region (Figure 2B and Figure 4A,B), Figure 2A identifies an area that is ordered in DENV and would be a good target of mutations during the development of an attenuated ZIKV. Again, since that region is ordered in DENV-2, order there could be tolerated by ZIKV.

## 5. Conclusions

There were two main ZIKV oddities discovered in this research. The first has to do with the relatively high disorder in the ZIKV M protein, which is at a level that is seldom found among other flaviviruses, with the exception of YFV. The other is the somewhat large variation in the disorder of ZIKV depending on the strain and lineage of the virus. These oddities are consistent with what we now know as characteristics of ZIKV. Just as other viruses (such as WNV and HIV-1) that have high outer shell (matrix/membrane) PIDs are able to penetrate vital organs, ZIKV can also penetrate the placenta and brain [12]. Furthermore, scientists have discovered that strains of the African lineage possess a greater potential to cause fetal morbidity. This fact correlates with the variations in the disorder levels of the ZIKV C protein. Disorder in the inner shell has already been associated with virulence and higher viral loads in other flaviviruses and other unrelated viruses, such as in the Ebola and Nipah viruses.

There are important implications for these findings. The results presented here could lead to the development of a new vaccine through the creation of attenuated strains resulting from more ordered C and M proteins. The regions for potential vaccine targets are disordered regions of the ZIKV M and C proteins that are ordered in other flavivirus species. Exploration of a ZIKV vaccine could include mutations in these areas. Other applications could include building a more comprehensive model that could anticipate the behaviors of new or little-known viruses. Lastly, the ability of ZIKV and YFV to penetrate vital organs as a result of shell disorder adds to a growing body of knowledge that encompasses a variety of viruses, such as HIV, that use such strategies to enter or to hide in hard-to-reach organs. An important potential application of this is in the field of cancer oncolysis, which involves the use of viruses to “attack” tumors, since both share the same pathways [68,69]. Viruses could be engineered to evade host immune systems that impede the effectiveness of therapeutic viruses. Moreover, since cancers in hard-to-reach organs such as the brain are often problematic for virotherapies and chemotherapies [14,70], a better understanding of the roles of shell disorder in the viral penetration of such organs will lead to improved therapies.

## Figures and Tables

**Table 1:** Percentages of intrinsic disorder (PIDs) of M and C shell proteins from *Zika virus* (ZIKV) strains with UniProt accession. Differences between the shell disorder in Asian and African strains are statistically significant (regression: *r*^2^ = 0.9, *F* = 15, *p* < 0.01; one-way analysis of variance (ANOVA) with C percentage of intrinsic disorder (PID) as the sole independent variable: *F* = 11, *p* < 0.05). Higher PIDs in C proteins were found in the African strains even as the PID levels could differ for C proteins in strains from the same lineage. The regression was performed using the assumption that the African strains have greater fetal morbidity than do the Asian strains [38,39,55].

Virus	Lineage	C PID ^d^	M PID ^d^	UniProt ID
Senegal 2001 Uganda 1947 (MR-766) ^a,^^b^	African strains	45.0 ± 0.1	28.0 ± 0.1	W8QFH2 Q32ZE1 ^a,b^
Uganda 1947 (MR-766) ^a,c^ Central African Rep 1976	African strains	37.0 ± 0.1	28.0 ± 0.1	A0A2S1KKZ9 ^a,c^ W0G5P7
Malaysia 1969 Colombia 2015 Brazil 2015	Asian strains	31.0 ± 0.1	29.0 ± 0.1	H8XX11 A0A127AM58 A0A0X9QZM7
Micronesia 2007	Asian strain	36.0 ± 0.1	29.0 ± 0.1	B3U3M3

^a^ Even though the samples were from approximately the same locations in 1947, they represented two separate isolates. While the two isolates are classified as the MR-766 strain, the shell disorder data pointed out a unique difference between these two isolates. ^b^ Isolate: ZIKV/*Macaca mulatta*/UGA/MR-766/1947-East_African. ^c^ Isolate: ZIKV/*Macaca mulatta*/UGA/MR-766-VEROE6-AC4-P12_07/1947. ^d^ The standard error is denoted by the prefix “±”.

**Table 2:** Mean PID of C and M proteins by flavivirus with UniProt accession codes (regression: *r*^2^ = 0.9, *F* = 18, *p* < 0.01; independent variables: C and M PIDs; dependent variable: CFR) [25].

Virus	CFR ^a,b,c^	C PID ^a,b^	M PID ^a,c^	UniProt ID
YFV	>50%	74 ± 1^d^	35 ± 1^d^	P03314, Q6DV88, Q1X881
TBEV-Fe	20–40%	68 ± 3	8.0 ± 0.1	G5CP55, P07720, K4P8A
TBEV-Si	2–3%	63 ± 1	6.0 ± 0.1	G8FGD9, G8FGD9
TBEV-Eu	1–2%	62 ± 2	6.0 ± 0.1	Q01299, P14336
DENV1-4	1.4 ± 0.8%	47 ± 5	11 ± 5	P14337, P09866, Q58Ht7
WNV	~0.4%	49 ± 4	9.0 ± 0.1	Q9EMB5, Q53AP1, P06935
ZIKV	<0.2%	35 ± 6	28.7 ± 0.5	P14336, Q32ZE1, H8XX11

^a^ A regression model using CFR as the dependent variable with M and C PIDs as independent variables yielded *r*^2^ = 0.8, *F* = 29, *p* < 0.01. Interaction between the two independent variables was seen as statistically significant. The PID are in percentages (%). ^b^ A regression model using CFR as the dependent variable with C PID as the independent variable yielded *r*^2^ = 0.3, *F* = 6, *p* < 0.05. ^c^ A regression model using CFR as the dependent variable with M PID as the independent variable yielded *r*^2^ = 0.4, *F* = 9, *p* < 0.05. Tick-borne encepthalitis virus(TBEV). ^d^ The standard error is denoted by the prefix “±”. YFV: yellow fever virus; WNV: West Nile virus.

## Supplementary Materials

The following are available online at https://www.mdpi.com/2218-273X/9/11/710/s1, Figure S1: Line graph showing correlations between fetal morbidity and shell disorder (M and C Protein PIDs).
