# Supplementary material for: Zika and Flavivirus Shell Disorder: Virulence and Fetal Morbidity
Source: Biomolecules. 2019 Nov 6;9(11):710. doi: 10.3390/biom9110710 (PMC6920988; doi:10.3390/biom9110710)
Supplement: Supplementary file 1 [file biomolecules-09-00710-s001.pdf]

Supplementary Data

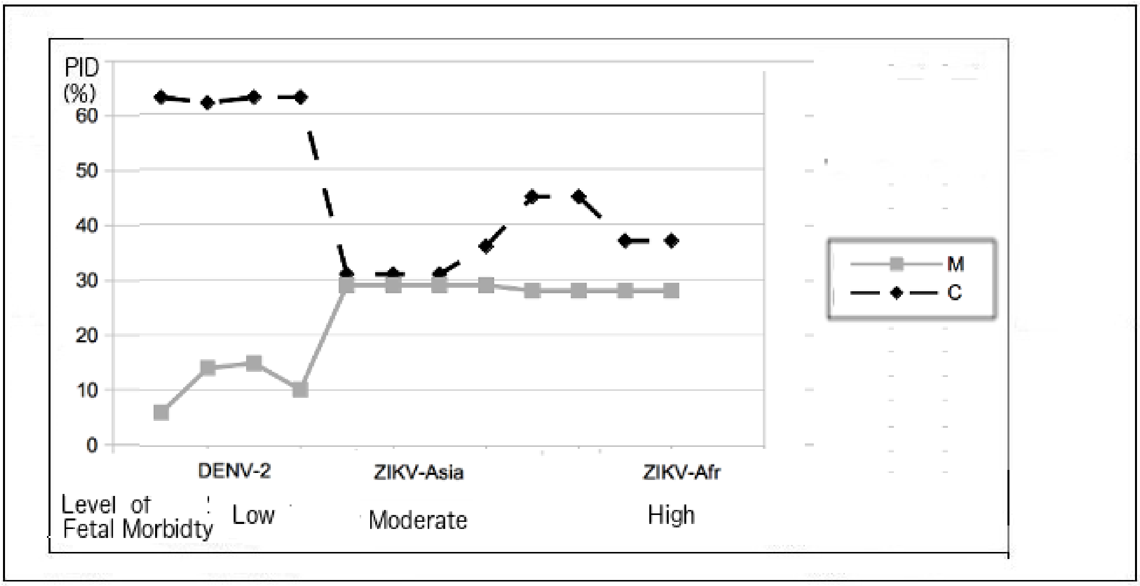

Figure S1. Line graph showing correlations between fetal morbidity and shell disorder (M and C Protein PIDs). (ZIKV/DENV2 Regression: $r^2 = 0.8$, $F = 16$, $p < 0.01$, independent variables: C and M PIDs. Poor correlation can be found when only C PID is used for both ZIKV and DENV-2 morbidity as DENV-2 has a high C PID and yet has a low fetal morbidity. When both M and PIDs are considered, a strong correlation can be found as DENV-2 has a low M PID, which could account for its low morbidity.
